# Pornography: How Does the Boomer Generation and Older Predict Others’ Viewing Time?

**DOI:** 10.1093/geroni/igaa057.998

**Published:** 2020-12-16

**Authors:** Katie Sakel, Joshua Grubbs

**Affiliations:** 1 Bowling Green State University, Gibsonburg, Ohio, United States; 2 Bowling Green State University, Bowling Green, Ohio, United States

## Abstract

The increase of exposure to online pornography has decreased the age of initial exposure to pornography. However, very little is known about the outcomes resulting from increased pornography exposure in the Baby Boomer generation and beyond. The current study asked what predictors were significant in individuals born in 1965 and earlier when predicting the perceived pornography viewing time for the average man and woman. To answer this question, a nationally representative population (N = 1073, 510 males) completed a web-based survey measuring the age of the participant, gender of the participant, self-directed sexual behaviors (“How frequently have you masturbated while viewing pornography alone?”), partner-directed sexual behaviors (“How frequently have you viewed pornography with a partner?”), a religiosity index (“How important is your religion?”), and the predicted perceived time that a woman and man watches pornography, Results showed that perceived time that the average man spent viewing pornography was significantly predicted by age of the participant, gender of the participant, self-motivated sexual behaviors and partner-motivated sexual behaviors. Religiosity was not a significant predictor. In the regression predicting perceived time that the average woman viewed pornography were age of the participant, self-motivated sexual behaviors, and partner-motivated sexual behaviors. Gender of the participant and religiosity of the participant were not significant predictors. Further research should expand this work to a lifespan perspective and longitudinal studies.

